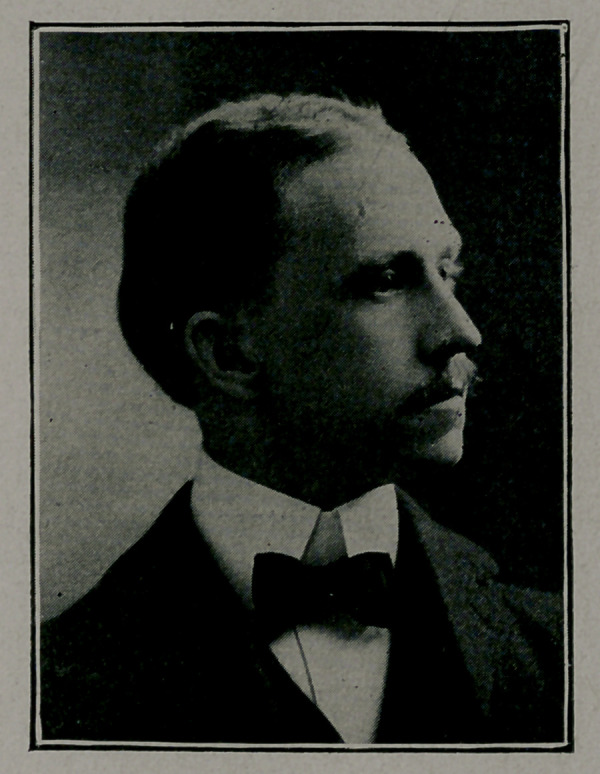# Editorials

**Published:** 1911-08

**Authors:** 


### EDITORIALS

The Business Office of the *Journal-Record* is 1 1-2 to 5 1-2 South Broad Street.
The Editorial Office is 1014-15 Century Building.
Address all Business Communications to *Journal-Record of Medicine*, 1 1-2 to 5 1-2 South Broad Street.
Make remittances payable *to The Journal-Record of Medicine.*

On matters pertaining to the Editorial and Original Communications, address Edgar G. Ballenger, M. D., Atlanta, Ga.
Reprints of Original Articles will be furnished at cost price. Requests for the same should always be made in THE MANUSCRIPT.

We will present, postpaid, on request, to each contributor of an original article, twenty (20) marked copies of *The Journal-Record of Medicine* containing such article.

### MR. BEAZLEY'S CRITICISMS.

In a recent issue of the Atlanta Constitution Mr. C. H. Beazley, of Leesburg, discusses the pellagra situation in Georgia and elsewhere from the standpoint of a layman, and seems somewhat peeved with the profession because they have not with unanimity proclaimed the etiology of pellagra.

He rises to inquire, I might say, with the late Alexander Pope

"Who shall decide when doctors disagree,
And soundest causuists doubt, like you and me?"

It is well known that many diseases have been treated with a considerable degree of success before their exact etiology was known. For instance, malaria was satisfactorily controlled before Laveran demonstrated the malarial plasmodium; diphtheria was fairly well managed long before the Klebs-Loffler bacillus was discovered; while numberless cases of gonorrhea were cured many moons before Neisser ever traced to his lair the gay and festive gonococcus.

It is, of course, highly important and eminently helpful in the treatment of any disease to know the etiology, but where that is not definitely settled, we can meet the therapeutic indications intelligently, while investigators are tracing from effect back to cause.

If Mr. Beazley's house were on fire, whether the causation was accidental or incendiary, would not prevent him from using recognized methods for extinguishing the flames. Later on, after the house had been saved, it would be interesting to discover the origin of the fire, so as to prevent a repetition in his house or others.

Just now many bright minds are focussed on this problem, and if our lay friends will be patient a little while, it is most probable that the prime causation of this distressing malady will be brought out positively and satisfactorily.

G. M. N.

### DEATH OF DR. JOSEPH N. LeCONTE.

It is with the deepest sympathy we record the death of our esteemed collaborator, Dr. Joseph Nisbet LeConte, who died Aug. 12, after a relapse of typhoid fever, which was complicated at the end with acute lobar pneumonia. Dr. LeConte is the first of our collaborators to die since the reorganization of the staff of the Journal-Record a number of years ago.

Dr. LeConte was born of a prominent Georgia family in 1813 and received a thorough scientific education, being graduated from Emory College and Bellevue Hospital Medical College. In 1898 after his graduation in medicine he entered the Jersey City Hospital where he remained as interne for 18 months. He then came to Atlanta where he entered practice in his chosen specialty, diseases of the gastro-intestinal tract. In 1906 he went to Berlin University for a course of study and had earned for himself an excellent reputation. Dr. LeConte attended well his own affairs and meddled not at all in those which did not concern him; he therefore enjoyed the respect and good-will of his fellow practitioners to a remarkable extent. In 1902 he was married to Miss Lillian King, of Atlanta. Two little daughters are left fatherless by his death. In behalf of the staff of the Journal-Record we extend our heartfelt sympathy to the bereaved family.

## Figures and Tables

**Figure f1:**